# Study protocol: One plus one can be greater than two—Ecological momentary assessment for Black prostate cancer survivors and partners

**DOI:** 10.1371/journal.pone.0255614

**Published:** 2021-08-09

**Authors:** Dalnim Cho, Kathrin Milbury, Yue Liao, Curtis A. Pettaway, Justin R. Gregg, Yisheng Li, Lorna H. McNeill

**Affiliations:** 1 Department of Health Disparities Research, The University of Texas MD Anderson Cancer Center, Houston, Texas, United States of America; 2 Department of Behavioral Sciences, The University of Texas MD Anderson Cancer Center, Houston, Texas, United States of America; 3 Department of Kinesiology, The University of Texas at Arlington, Dallas, Texas, United States of America; 4 Department of Urology, The University of Texas MD Anderson Cancer Center, Houston, Texas, United States of America; 5 Department of Biostatistics, The University of Texas MD Anderson Cancer Center, Houston, Texas, United States of America; PLOS ONE, UNITED STATES

## Abstract

Given that romantic partners play a pivotal role in patients’ survivorship period, integrating partners into survivorship care and broadening the focus of behavioral interventions from the individual (survivor) to the survivor-partner dyad may make healthy lifestyle behaviors more easily adopted and potentially maintained. Understanding the role of dyadic processes in Black survivors is particularly important because their lifestyle behaviors are poor and they have higher cancer-specific and all-cause mortality. To develop an effective dyadic lifestyle behavior intervention for Black survivors, micro-level investigations of interactions between Black survivors and their partners are necessary to pinpoint how survivors and partners facilitate or hinder each other’s lifestyle behaviors in their natural, everyday lives. Accordingly, the objective of the present study is to fill these gaps using ecological momentary assessment to eventually develop more effective lifestyle interventions for Black prostate cancer (PCa) survivors and partners. A total of 120 dyads (i.e., 240 individuals) who are Black adult survivors diagnosed with non-metastatic PCa and their romantic partners will be asked to complete four assessments per day for 14 consecutive days on a smartphone after an initial retrospective survey. Over the 14 days, participants will be asked to complete a brief survey regarding their lifestyle behaviors (physical activity, sedentariness and eating behaviors), contexts of lifestyle behaviors, stress, and coping. Physical activity and sedentary behavior will be assessed via accelerometer; eating behaviors will be assessed with the Automated Self-Administered 24-hour Dietary Assessment Tool. After completing the 14-day assessment, participants will be asked to complete a final retrospective survey. Results of the proposed study will inform the rigorous development of a theory-based dyadic lifestyle intervention in this vulnerable survivorship population with the ultimate goal to improve overall survival and reduce morbidities (for survivors) and reduce cancer incidence (for partners).

## Introduction

Prostate cancer (PCa) is the most commonly diagnosed cancer in American men [1], but the burden of PCa is the highest among Black men [2, 3]; compared with non-Hispanic White Americans, Black PCa patients and survivors are 140% more likely to die [3] and report lower quality of life [4–6]. Healthy lifestyle behaviors such as physical activity, low sedentary behaviors, and healthy eating behaviors are especially important for PCa survivors, given that many men are more likely to die from competing causes (e.g., cardiovascular disease) than PCa itself [7], which can be prevented by healthy lifestyle behaviors [8–10]. Indeed, studies have found that healthy lifestyle behaviors can reduce mortality [11–17], delay disease progression [13, 18], and enhance quality of life among PCa survivors [11, 19, 20].

While many lifestyle interventions have been developed for cancer survivors [21] and are effective for behavioral changes, their effects are small-to-medium [22], mechanisms of behavioral changes are unclear [21, 22], and long-term behavioral changes fail to occur [21, 23, 24]. Furthermore, Blacks are seriously underrepresented in these interventions and are not sufficiently informed about available behavioral change resources [25]. Innovative lifestyle interventions that have the high potential for increasing healthy lifestyle behavior adoption and maintenance, therefore offer the potential to reduce cancer health disparities among Black PCa survivors, are urgently needed.

### Interdependence between Black PCa survivors and caregivers

Cancer is often considered a “we-disease” [26]; survivors and partners react to cancer as an interdependent system in which they influence each other’s health. PCa strongly impacts not only survivors but also their partners [27–30], who are often reported as the primary source of support for Black PCa survivors [31]. Thus, it may be integral to include partners in Black PCa survivorship care and broaden the focus of lifestyle interventions from the individual to the survivor-partner dyad [32]. However, results from existing dyadic lifestyle interventions for survivors and partners (or caregivers) do not lend strong support for targeting the dyad as a single unit of care [33–35], although none of them specifically targeted Black PCa survivors. At present, no evidence exists that dyadic lifestyle interventions are superior to individual-only ones for couples dealing with illness [36, 37].

We argue that these unexpected results are partly due to the fact that these dyadic lifestyle interventions were simply adding partners to the existing individuals-only interventions and did not take on a truly dyadic perspective. In order to maximize the impacts of the dyadic lifestyle interventions, we need to address factors (beyond the exchange of social support) that will lead to greater behavioral changes in survivors than survival-only interventions and will promote behavioral changes in both survivors and partners. Nevertheless, the characteristics of Black PCa survivor-partner dyads that facilitate these behavioral changes are completely unknown. Before implementing another dyadic lifestyle intervention, micro-level investigation of interactions between survivors and partners is necessary to pinpoint how Black PCa survivors and partners facilitate or hinder each other’s lifestyle behaviors in their natural, everyday lives.

### Stress, coping, and lifestyle behaviors in the context of Black PCa survivorship

Stress can substantially influence poor lifestyle behaviors [38–40], but it is an understudied characteristic for Black PCa survivors’ lifestyle behaviors. Importantly, beyond shared stressors (e.g., concerns about cancer recurrence/progression), survivors and partners will encounter various stressors outside the relationship individually in their everyday lives (e.g., daily discrimination, conflicts at work, other social obligations), which can spill over into their relationship. Thus, individual stress can also influence both couple members’ lifestyle behaviors [41].

**Fig 1:**
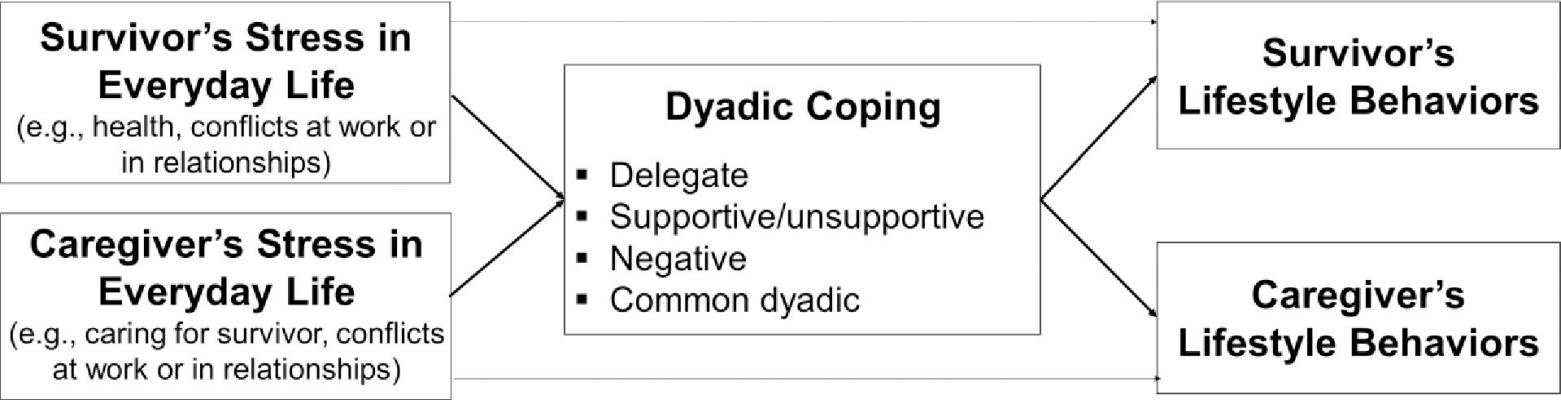
Conceptual model.

Dyadic-level theories such as the systemic-transactional model [42, 43] posit that partners cope both individually and collectively as a unit to regulate stressors. Dyadic coping is a unique concept in the theories and indicates that couples respond to stressors as interpersonal units rather than as individuals in isolation [44]. Dyadic coping can be either positive or negative, and includes *supportive/unsupportive dyadic coping* (e.g., providing practical help, showing disinterest), *delegated dyadic coping* (e.g., taking on things that the partner normally does), *negative dyadic coping* (e.g., mutual avoidance) and *common dyadic coping* (e.g., joint problem solving). Thus, dyadic coping goes beyond the exchange of social support [43]. Although supportive dyadic coping may enhance the stressed partner’s lifestyle behaviors, common dyadic coping is likely to be associated with both members’ positive behavioral change (see Fig 1 for our conceptual model). To date, no studies have tested the impacts of dyadic coping on lifestyle behaviors among Black PCa survivors and partners.

### Ecological Momentary Assessment (EMA) of stress, coping, and lifestyle behaviors research

EMA is a real-time data capture method that can be used to assess transient influences in individuals’ every day, natural settings [45, 46]. Specifically, effects of stress and coping on lifestyle behaviors may act on a shorter time scale: Stress and dyadic coping may vary across days of a week (i.e., within-person) or even across times of a day (i.e., within-day) [47–49], and these time-varying factors may explain the within-person and within-day variations in lifestyle behaviors [50–55]. For example, after work, a partner of a PCa survivor may be exhausted and prepare fast food for family dinner, and/or may decide to stay at home instead of walking outside together. Thus, global, aggregated measures of stress and coping at the between-person level (e.g., ‘How often were you stressed in the *last month*?’), which are often assessed in studies on stress and coping, may neglect the subtle and dynamic changes in lifestyle behaviors during the course of everyday life [56]. Also, as between-person level associations do not necessarily reflect those at the within-person or within-day levels [51, 53, 57, 58], data captured by EMA at micro timescales may provide additional insights [46]. Furthermore, EMA can identify the social and physical contexts of lifestyle behaviors as they occur. Considering these contexts is essential in the design of interventions to plan who should deliver the interventions and where the interventions should be situated [25].

### The present study

The objective of the present study is to fill the gaps in knowledge using data from EMA to ultimately develop more effective lifestyle interventions for Black PCa survivors and partners (i.e., interventions that promote both survivors’ and partners’ lifestyle behaviors and result in greater behavioral change). Our long-term goal is to develop an innovative Just-In-Time-Adaptive-Intervention (JITAI) that provides the right treatment to the right person at the right time [59], which is feasible for Black PCa survivors and their partners to undertake and for clinic or community facilities to provide.

Our specific aims are to examine temporal associations between dyadic coping and lifestyle behaviors (*Aim 1*). We hypothesize that common dyadic coping will be associated with both members’ positive behavioral changes. We also aim to identify the social and physical contexts in which lifestyle behaviors occur/co-occur (*Aim 2*). We will examine the physical contexts (e.g., where survivors and partners engage in health behaviors together or separately) and social contexts (e.g., who they engage in health behaviors with). Finally, we aim to investigate potential moderators for the associations between stress and dyadic coping (*Exploratory Aim*). We expect that the associations between stress and dyadic coping will depend on factors such as relationship quality, time since cancer diagnosis, type of stress, and home/neighborhood environments.

## Methods

### Participants

Survivors are eligible if they: (a) are Black adults; (b) had stage 0-III PCa; (c) completed adjuvant therapy (i.e., chemo and/or radiation therapy); (d) live together with a current partner/spouse; (e) do not need physical assistance (e.g., wheelchair, cane); (f) have a smartphone; (g) can read, write, and speak English; and (h) currently are not participating in a lifestyle behavior or weight management program. PCa patients managed utilizing active surveillance will also be included. Survivors will be excluded if they have a prior history of other cancer or have metastatic cancer. Partners are eligible if they: (a) are adults; (b) do not have serious medical conditions (e.g., cancer, congestive heart failure, stroke, and dementia); (c) have a smartphone; (d) can read, write, and speak English; and (e) currently are not participating in a lifestyle behavior or weight management program. The dyad can be either married or unmarried and same-sex or heterosexual. If smartphone ownership is the only reason making either survivor or partner ineligible, the study team will lend a smartphone and the participant will be enrolled. This study was approved by the University of Texas, MD Anderson Cancer Center (MDACC) Institutional Review Board (protocol #: 2019–0579) on September 19, 2019.

### Procedures

#### Recruitment

We will implement both active and passive recruitment strategies to maximize recruitment. For the active strategy, we will work closely with urologists to identify potentially eligible participants before their clinic visit at MDACC and at Lyndon B. Johnson Hospital (LBJ) of the Harris Healthcare System. During their clinic visit, urologists and their clinical staff will inform eligible patients about this study and they will be approached by our research staff. Research staff will explain the purpose of the study and interested persons will be further screened. If survivors are interested in the study, but partners are not present during the clinic visit, the research staff will ask permission to contact the partners via phone to screen their eligibility. We will also recruit participants through The Center for Community-Engaged Translational Research (CCETR), a shared research resource at MDACC. This study will be imparted to Black churches, PCa support groups, and local/institutional health fairs/events through CCETR. For the passive strategy, we will use the MDACC Tumor Registry and the State of Texas Cancer Registry to identify potentially eligible individuals. An invitation letter will be sent via postal mail and then they will be followed-up by phone from the research staff. Thus, the active strategy will help us recruit partners and recent/new survivors and the passive one will recruit existing survivors.

#### Initial assessment

Research staff will obtain signed informed consent from both survivors and partners. Participants will complete their initial assessment at MDACC Behavioral Research and Treatment Center (BRTC) or churches/community centers that are close to their home/workplace to reduce travel-related burdens. During this visit, the survivor-partner dyads will be asked to complete a self-administered survey via REDCap to assess demographics and retrospective lifestyle behaviors. At the end of this visit, research staff will install the EMA application to each participant’s smartphone and provide instructions on how to use it. Also, all participants will be provided with a blind accelerometer (Actigraph GT3X+ or GT3X-BT) and be asked to wear it on their waist during waking hours (except bathing and swimming) on their right hip for 14 days and to engage in their daily routines as normal. EMA and accelerometer wear will start the day after the initial visit. After 14 days, they will visit MDACC BRTC or their local church/community center to complete the survey (final survey) and return the accelerometer.

If participants are unable to make the in-person visit (due to safety concerns regarding COVID-19, time conflicts, transportation issues), they will be asked to complete assessments remotely. After completing the REDCap survey and receiving accelerometers (delivered by the postal service), participants will be instructed on how to wear the accelerometers and download the EMA app via video conferencing. If appropriate, the study team will lend a smartphone to complete the EMA via postal mail and the participant will return the device after completing the 14-day EMA; the study team will provide stamped envelopes.

#### EMA procedures

A smartphone-based EMA application will be developed by Mobile Health, a shared resource at the NCI-designated Stephenson Cancer Center. All participants will be asked to complete 4 assessments per day, for 14 days, at fixed times (7:30am, 11:30am, 3:30pm, 7:30pm) to capture lifestyle behaviors, stress and coping throughout the day (morning, midday, afternoon, and evening). During each assessment, participants will be asked to complete a brief (~5 minute) survey. Couples will complete all assessments individually. Each assessment will be audibly and visually cued. If the participant does not respond to the initial prompt, two reminder prompts will occur with a 10-minute interval. If the participant fails to respond to the third prompt, the assessment will become inaccessible and will be counted as missed, and this information will be coded in the database. Participants will be told to ignore the prompts if they occur at inconvenient times (e.g., driving, bathing, business meeting). The actual time between initial prompting and assessment completion will be tracked by the EMA application for later analysis. Any assessments not completed during the allotted time (i.e., within 30 minutes) will be recorded as missed. This ensures that entries are not mass entered only at certain times of the day, such as at the end of the day, and the data being captured reflects the momentary nature of the assessment. The application works either online or offline and participants will not be able to self-initiate any of the EMA assessments. After each entry, EMA data will be synced to a secure web-server, where investigators can monitor compliance. If the smartphone is not connected to the internet, the data will be stored on the phone first, then be synced to the server when there is internet connection.

To enhance study participation and compliance, participants will receive a text message every night after their final assessment that includes a brief “thank you” note for study participation, a reminder to complete assessments for the remaining days, and the research team’s contact information for any questions and troubleshooting.

Each participant will receive $20 to complete each initial and final survey (plus a parking validation, if appropriate) and up to $100 to complete the 14-day assessments and wear accelerometers. Reimbursement for the EMA procedures will be prorated based on compliance (≥90% completion = $100; 75–89% = $90; 60–74% = $70; 50–59% = $60; <50% = no compensation). Participants will receive $10 to complete each nutrition assessment (twice/week with the 24 h recall nutrition assessment [60]). Thus, a participant can be compensated up to $180.

### Measures

#### Baseline demographic/medical factors and retrospective measures

Both survivors and partners will be asked to report demographic and health information, such as age, level of education and household income, length of romantic relationship, health insurance, and existence of chronic illnesses (e.g., diabetes, hypertension). Tumor characteristics will be self-reported (survivors only), but will also be obtained from medical records, if recruited from MDACC and LBJ. At both initial and final surveys, we will retrospectively assess relationship satisfaction (Dyadic Adjustment Scale-4) [61], stress over the past week (Multidimensional Stress Questionnaire for couples; MSQ-C) [62], dyadic coping (Dyadic Coping Inventory; DCI) [63, 64], and lifestyle behaviors (physical activity and sedentary behavior [65] and eating behaviors [66]), as relationships at this macro, longer-term level may differ from those at the micro levels. The MSQ-C is a 30-item instrument (1 = *not at all stressful*, 5 = *very stressful*) reflecting a range of stressors external (e.g., problems getting along with fellow workers) and internal to the dyad (e.g., health of your partner). The DCI is a 37-item instrument (1 = *very rarely*, 5 = *very often*) designed to measure dyadic coping. We will also assess environmental characteristics for lifestyle behaviors that may provide useful information about barriers and facilitators to lifestyle behaviors [67–69] such as perceived home environment (e.g., availability of healthy and unhealthy foods/exercise equipment at home [70, 71]) and perceived neighborhood walkability [72, 73] and food environments (e.g., fast food availability) [74]. At the final assessment, a brief exit survey will be conducted to understand the level of difficulty and stress of completing EMA, barriers to responding to prompts, and future recommendations.

#### EMA measures

To decrease participants’ burden, reduced items will be used instead of a full scale. Participants will be asked to respond to questions within a certain time frame such as “since waking up this morning (first assessment of a given day),” or “since your last assessment (all other three assessments).” Stress will be assessed with five items adapted from the MSQ-C [62] that cover health, social, finances, and job domains. For example, “Hassles are irritants that can range from minor annoyances to fairly major pressures, problems, or difficulties. Since waking up this morning, have the following hassles happened to you (y/n)?”: *1) the possibility of your (your partner’s) cancer recurrence/progression*, *2) conflicts with your partner*, *3) conflicts with other family members*, *friends*, *co-workers*, *and others*, *4) financial issues (e*.*g*., *money for basic necessities*, *money for emergencies*, *retirement)*, *and 5) work-related issues (e*.*g*., *challenging work*, *job dissatisfaction) [each item in separate screens])*. If participants respond yes, they will be asked to report its severity on each stressor (1 = *not at all stressful*, 5 = *very stressful*). Unless participants reported 1 (*not at all stressful*) to the specific stressor, dyadic coping will be assessed with eight items (two items for each coping) from the DCI [63, 64] with a stem *“what did your partner do when you were stressed*?*”* (1 = *very rarely*, 5 = *very often*): supportive/unsupportive (e.g., ‘helped me to see stressful situations in a different light’), delegate (e.g., ‘took on things that I normally do in order to help me out’), negative (e.g., ‘did not take my stress seriously’), and common dyadic coping (e.g., ‘we tried to cope with the problem together and search for solutions’). For self-reported lifestyle behaviors, physical activity (e.g., brisk walking, gardening, dancing, exercise/sports) and sedentary behavior (e.g., watching TV, on the computer/internet, riding in a car/transportation) occurrence and time spent will be asked. Moderate-to-vigorous physical activity (≥3 MET) and sedentary hours will be calculated. Dietary behaviors such as eating chips/fries, sweets, and fruits & vegetables and drinking regular soda will be assessed (yes/no). Finally, the social and physical contexts of lifestyle behaviors will be assessed (only when lifestyle behaviors occur) with one item each for each lifestyle behavior, for example, *‘Who were you with when you exercised*?*’* and *‘Where were you when you ate sweets*?*’*.

#### Lifestyle behaviors

All participants will be asked to wear a blinded accelerometer (Actigraph GT3X+ or GT3X-BT) on their waist during waking hours (except bathing and swimming) on their right hip for the 14 days. The Actigraph records counts of physical activity throughout the day in user-set “epochs” and steps per minute. In line with the national surveillance data [75], moderate-to-vigorous physical activity and sedentary behavior thresholds will be ≥ 2,020 counts/min and < 100 counts/min [76], respectively.

Dietary intake will be assessed with the Automated Self-Administered 24-hour (ASA24) dietary assessment tool [60] that establishes healthy eating index. It is a reliable and valid measure of diet quality to assess compliance with the Dietary Guidelines for Americans [77]. It contains 13 components (e.g., fruits, vegetables, added sugars, saturated fats) that sum to a total maximum score of 100 points indicating the set of foods reported is in line with the Dietary Guidelines recommendations. During the initial survey, participants will be asked to select two days [a weekday (Monday-Thursday) and a weekend day (Friday-Sunday)] per week (i.e., 4 assessments total) on which they will be contacted by our research staff over the phone to complete the nutrition assessment.

### Data analytic plans

We will first conduct extensive descriptive analyses on the data collected at baseline, across the 14-day period (e.g., by day and by time of day) and at the final assessment. Descriptive statistics, e.g., means, standard deviations (SDs), and ranges for continuous measures, and frequencies and proportions for categorical variables, along with 95% confidence intervals (CIs) for the means will be calculated. Preliminary associations among variables (e.g., stress, coping, and lifestyle behaviors) will be assessed at specific time points of interest using Pearson and Spearman correlation coefficients, t-tests, analysis of variance (ANOVA), chi-squared or Fisher’s exact tests, where appropriate.

For Aim 1, there are two primary lifestyle behavior outcome variables: physical activity (count/minute; continuous) and nutrition (ate fruits/vegetables or not during the last EMA measurement to the current time point; binary). Each lifestyle behavior is measured for both survivors and partners and at each time point (a total of four within a day), when applicable. The primary predictor variable of interest is common dyadic coping, again measured for both survivors and partners. Our primary analysis will treat the common dyadic coping as a continuous predictor (with a score of 1–5). However, other methods will be explored by treating it as categorical (such as above or below certain thresholds).

Our analysis will mainly use generalized linear mixed-effects models (GLMMs), which can account for binary outcomes with a canonical (logit) link function and the within-subject (possibly differentially for within-day and across-day) correlations using random effects and a variety of repeated measures correlation structure, as appropriate. The GLMM will reduce to a linear mixed model (LMM) for the continuous physical activity outcome. A variance-stabilizing transformation such as the square-root or log transformation may be applied to the continuous physical activity outcome, as appropriate. When both the survivors’ and partners’ outcomes are analyzed, the within-dyad correlation will be accounted for using random dyad effects. The random effects and repeated measures correlation structure will be selected using the Bayesian information criterion (BIC).

To assess the association between common dyadic coping of partner or survivor and each lifestyle behavior of the survivor or partner (yielding two associations of interest for each lifestyle behavioral outcome of each of the survivor and partner, and thus, eight associations of interest across the four lifestyle behavior outcomes), we will construct two new variables corresponding to each common dyadic coping variable for each individual: 1) the mean across all common dyadic coping measurements within the individual, representing an “average” individual coping level; and 2) the deviation of situation-specific common dyadic coping from the individual-specific average, representing a within-subject level coping deviation. Both variables, in place of the one original common dyadic coping variable, will be included in the model and tested for their associations with the lifestyle behavioral outcome. This approach allows us to test for both the between-subject and within-subject effects of common dyadic coping on a lifestyle behavior, which is made possible given the intensive EMA measures [78]. To control the overall type I error rate for testing for these 16 primary associations of interest (eight associations for each of the two newly constructed predictors of interest), we will use a two-sided 0.05/16 = 0.0031 significance level with a Bonferroni adjustment. Secondary analyses will be conducted for the binary outcomes of moderate-to-vigorous physical activity and sedentary behaviors using similar GLMMs. Secondary associations will be tested between the lifestyle behaviors and the other predictors of interest, specifically, the three other dyadic coping variables (supportive/unsupportive, delegate, negative) and stress (average of the five stress items), using similar GLMM analyses. The same analyses will also be conducted to evaluate the associations between stress and coping. All tests will use a two-sided 0.0031 significance level.

However, significance based on the secondary analyses will be interpreted as hypothesis generating, given that the overall type I error rate is not adequately controlled for in these secondary analyses. Similar analyses at the within-day level (i.e., including both a day-specific predictor average variable and the deviation from the day-specific average as predictors in the model) will also be conducted. All analyses described above assume the lifestyle behaviors and predictors of interest are measured during the same time intervals of day for each individual. Additional exploratory analyses will examine lagged associations, i.e., the effects of the predictors measured in the previous time interval (e.g., in the morning) on the lifestyle behavior outcome in the current time interval (e.g., at noon); or alternatively, common dyadic coping could be measured based on potential interactions between the survivor and partner during the past 24 hours, given that such interactions may not occur as often as in four-hour time intervals during each day. All these exploratory analyses are possible, again given the intensive EMA measures.

We will perform the above-mentioned analyses with and without controlling for the baseline covariates of interest, including demographics (e.g., age, level of education and income) and physical activity or nutrition variables. The association between common dyadic coping and health eating index (0–100), which is measured only four times for each individual, will be conducted by using summarized measures of common dyadic coping for the corresponding periods in which the health eating index is calculated. For example, if the health eating index is calculated for a whole week that includes all work days, then the summaries of common dyadic coping may be the mean and/or standard deviation of common dyadic coping measured during the week (and across days). To derive such summary features of common dyadic coping, we will follow the methods of Cofta-Woerpel and colleagues [79].

For Aim 2, descriptive analysis (e.g., frequency) will be conducted to examine the places (e.g., home, neighborhood) in which the lifestyle behaviors occurred and the number of lifestyle behaviors engaged alone and with others (especially with partner). In addition, the contexts (days of a week and times of a day and place) in which survivors and partners engaged in lifestyle behaviors together will be investigated. All analyses for assessing Aim 2 will be exploratory in nature.

For the exploratory aim (moderation effects of the relationship quality, time since cancer diagnosis, type of stress and home/neighborhood environments) will be examined in the associations between stress and dyadic coping by adding to and testing for their interaction effects with the predictors of interest. Significance at a two-sided 0.05 level will be deemed interesting findings, due to the exploratory nature of this aim.

### Sample size and power calculation

Our sample size justification is based on testing for the 16 primary associations of interest, i.e., each newly constructed common dyadic coping variable of a partner or survivor with each lifestyle behavior of the survivor or partner, each at a two-sided 0.0031 significance level. That is, we will test the effects of both the between-subject variable (the average within-subject common dyadic coping) and the situation-specific deviation of the common dyadic coping from the subject-specific average. For the between-subject variable, by assuming conservatively a 10% loss to follow-up of individuals and an additional attrition of 25% lifestyle behavior or EMA measurements for those who are not lost to follow-up, and an ICC of 0.5, a 0.0031 two-sided Fisher’s z test of the null hypothesis that the Pearson correlation coefficient r = 0 between common dyadic coping and physical activity, will have 80% power to detect an r of 0.295 (small to medium effect) when the sample size is 159 (nQuery 7.0). The effective sample size is calculated by accounting for the variance inflation factor (= 120×0.9×0.75×14×4/(1+(14×4–1)×0.5)). If the ICC is 0.2, the sample size is 378, resulting in a detectable correlation of 0.195 with 80% power. To test the association between a common dyadic coping variable and a nutrition outcome (binary), a logistic regression analysis (assuming an approximately normally distributed common dyadic coping variable) with a sample size of 119 observations (i.e., 3/4 of the effective sample size of 159, assuming at least three out of the four nutrition measurements are available) achieves 80% power at a 0.0031 significance level to detect a change in the probability of eating fruits/vegetables from the value of 0.300 at the mean of common dyadic coping to 0.478 when the common dyadic coping is increased to one standard deviation above the mean. This change corresponds to an odds ratio of 2.138 (PASS 2005). When the ICC is 0.2, the corresponding detectable odds ratio will be 1.637 under an effective sample size of 378. The power to detect the within-subject effect of the variable of deviation of common dyadic coping from its individual-specific average is generally higher than detecting the same level of between-subject effect. Thus, our proposed sample size will yield adequate power to detect the 16 primary associations of interest, while allowing reasonable assessment of the secondary and exploratory associations of interest.

## Discussion

The proposed study seeks to shift current individual-focused (either survivors or caregivers) research and clinical paradigms to relationship-focused ones. To our best knowledge, this is the first study that explicitly investigates a unique, theory-based concept, dyadic coping, which is beyond social support for Black PCa survivors’ and partners’ lifestyle behaviors. Results of this study are expected to lead to the development of a dyadic lifestyle intervention that addresses stress and dyadic coping that may have higher effects for behavioral changes compared to individual-only interventions. This is also the first micro-longitudinal study that investigates in-depth interpersonal processes between Black PCa survivors and their partners that are essential for the future intervention development. Despite the significant roles of partners in the lives of Black men [80], very few studies including partners of Black PCa survivors exist. Thus, the proposed study may add to the literature in that it aims to find factors that maximize the roles of partners in supporting Black cancer survivors. Furthermore, this is also the first study that investigates the natural, real-world contexts in which lifestyle behaviors occur among Black survivors and partners, which enhances the ecological validity of the study [56]. Understanding these contexts may inform dissemination and implementation plans ahead. In the long-term, if the developed intervention is proven effective and widely disseminated, targeting survivor-partner dyads may have significant public health impacts by decreasing the likelihood of developing cancer in partners (primary prevention) and decreasing likelihood of morbidity/mortality in Black PCa survivors (tertiary prevention).

### Limitations

Because partners are often primary caregivers among Black PCa survivors [31] and relationship dynamics may significantly vary across different types of dyads (e.g., father survivor-daughter caregiver dyads), this study intentionally targets partner caregivers. Thus, results of this study will not be generalizable to a Black PCa survivor who does not have a partner or whose primary caregiver is not his partner. Participants’ burden to complete four assessments for 14 days may be high, which can lower compliance. However, a previous 14-day EMA study conducted among cancer survivors, which included more assessments than the present study (6 times per day), showed a good compliance rate (76% completion) [81]. While it is highly likely the four daily assessments will not be too cumbersome, future research will be needed to obtain an acceptable frequency of assessments per day in this population.
